# Adequate, questionable, and inadequate drug prescribing for older adults at the end of life: a European expert consensus

**DOI:** 10.1007/s00228-018-2507-4

**Published:** 2018-06-23

**Authors:** Lucas Morin, Marie-Laure Laroche, Davide L. Vetrano, Johan Fastbom, Kristina Johnell

**Affiliations:** 1 Aging Research Center, Karolinska Institutet, Tomtebodavägen 18A, 171 77 Stockholm, Sweden; 2 Centre de pharmacovigilance et de pharmaco-épidémiologie, Centre Hospitalier Universitaire de Limoges, Limoges, France; 3 Faculté de Médecine, Université de Limoges, Limoges, France; 4 Department of Geriatrics, Catholic University of Rome, Rome, Italy

**Keywords:** Palliative care, Older adults, Drug prescribing, Quality of care, Inappropriate prescribing

## Abstract

**Background:**

Clinical guidance is needed to initiate, continue, and discontinue drug treatments near the end of life.

**Aim:**

To identify drugs and drug classes most often adequate, questionable, or inadequate for older people at the end of life.

**Design:**

Delphi consensus survey.

**Setting/participants:**

Forty European experts in geriatrics, clinical pharmacology, and palliative medicine from 10 different countries. Panelists were asked to characterize drug classes as “often adequate,” “questionable,” or “often inadequate” for use in older adults aged 75 years or older with an estimated life expectancy of ≤ 3 months. We distinguished the continuation of a drug class that was previously prescribed from the initiation of a new drug. Consensus was considered achieved for a given drug or drug class if the level of agreement was ≥ 75%.

**Results:**

The expert panel reached consensus on a set of 14 drug classes deemed as “often adequate,” 28 drug classes deemed “questionable,” and 10 drug classes deemed “often inadequate” for continuation during the last 3 months of life. Regarding the initiation of new drug treatments, the panel reached consensus on a set of 10 drug classes deemed “often adequate,” 23 drug classes deemed “questionable,” and 23 drug classes deemed “often inadequate”. Consensus remained unachieved for some very commonly prescribed drug treatments (e.g., proton-pump inhibitors, furosemide, haloperidol, olanzapine, zopiclone, and selective serotonin reuptake inhibitors).

**Conclusion:**

In the absence of high-quality evidence from randomized clinical trials, these consensus-based criteria provide guidance to rationalize drug prescribing for older adults near the end of life.

**Electronic supplementary material:**

The online version of this article (10.1007/s00228-018-2507-4) contains supplementary material, which is available to authorized users.

## Introduction

Near the end of life, drug treatments should focus on *palliative* goals of care by gradually reducing disease-oriented therapy and prioritizing symptom relief and comfort [1–3]. Yet, polypharmacy is commonplace during the final months of life of older adults, fueled not only by the surge of analgesics and other symptomatic drugs but also by the continuation of medications prescribed for the long-term prevention of chronic diseases [4–7]. This highlights the need to rationalize drug prescribing near the end of life [8, 9].

Drugs are usually considered as potentially inappropriate when the risk of harmful effects outweighs their expected benefit, or when there exist a safer, better tolerated, or more effective alternative [10]. Several assessment tools have been developed over the past two decades to help prescribers identify potentially inappropriate drugs, including the Beers and the STOPP/START criteria [11, 12]. However, these tools are not designed to evaluate the quality of drug prescribing in the context of end-of-life care. Drug classes considered as potentially inappropriate with regard to their risk of side effects (e.g., opioids, benzodiazepines, scopolamine) may be indicated for the management of distressing symptoms during the final weeks of life. Conversely, drugs appropriate for older adults in general may be of little benefit and present a higher risk of harmful effects near the end of life. Holmes et al. have therefore proposed a prescribing model that goes beyond the pharmacological focus of existing criteria [13]. They suggest that physicians should consider stopping otherwise appropriate medications when the time needed to achieve a clinically significant outcome is longer than the remaining life expectancy, or when the treatment target is no longer aligned with the preferred goals of care.

Deprescribing has gained considerable attention in geriatric medicine [14, 15]. However, there is limited evidence to assess the benefits and risks of discontinuing drug treatments in older adults with limited life expectancy [16, 17]. Older adults with advanced illness and multiple chronic diseases are typically excluded from randomized controlled trials and well-designed prospective cohort studies are scarce. Clinical guidance must therefore mostly rely on expert consensus. Yet, until now most initiatives have focused on a single disease (e.g., dementia [18], cancer [19–21]), or have adopted a broader perspective encompassing all frail older adults without specifically targeting the final months of life (e.g., STOPPFrail [22], NORGEP-NH [23]). While the former have a limited generalizability, the latter may prove difficult to apply in real-world clinical practice because of the considerable uncertainty that comes with estimating the life expectancy of frail older adults. Survival predictions made by physicians in routine clinical practice are often over-optimistic, and are notoriously unreliable when the actual survival exceeds a few months or weeks [24–26]. One way to reduce this prognostic uncertainty is to focus on a shorter period where end-of-life care needs are easier to ascertain.

The purpose of this study was to develop a consensus about prescription drugs that are most likely *adequate*, *questionable*, or *inadequate* for people aged 75 years or older with an estimated life expectancy of ≤ 3 months. Our goal was thereby twofold: (i) to provide guidance for prescribers caring for older adults nearing the end of life and (ii) to develop a tool that could be used to assess the quality of drug prescribing in pharmacoepidemiological studies based on routinely collected data.

## Methods

### Selection of panelists

The consensus panel consisted of European clinicians and researchers with in-depth expertise of drug prescribing in older adults at the end of life. We only included healthcare professionals with clinical experience in either palliative medicine, geriatrics, family medicine, or pharmacology. Potentially eligible participants were identified through a literature review, selecting principal investigators and principal authors of relevant original studies on the topic. To broaden the panel of experts, we also asked key informants to provide names of potential participants. Age, gender and occupational and geographical balance were ensured during the selection of panelists. Eligible candidates were emailed an invitation presenting the study aims and the Delphi process. Participants who agreed to participate were required to sign an electronic consent form and were informed that they could withdraw from the survey at any time. No financial incentive was provided.

### Selection of criteria

We conducted a systematic review of the literature updating the findings from Todd et al. [1] to identify therapeutic classes or individual drugs considered as potentially adequate or inadequate for use in the context of end-of-life care. A preliminary list of 49 drug classes was developed by a research team consisting of two clinical pharmacologists, one pharmacist, and one geriatrician. We included items at the pharmacological subgroup level of the Anatomical Therapeutic Chemical (ATC) classification rather than at the individual drug level (e.g., “A02B Drugs for peptic ulcer and gastro-oesophageal reflux disease” instead of “A02BC01 omeprazole”). Our study focused on drug treatments, and did therefore not include other types of medical treatments such as herbal drugs, mechanical ventilation, or tube feeding. However, because blood transfusion is a therapeutic alternative to drug treatments in the management of anemia and thrombocytopenia, it was also included.

### Delphi process

The candidate set of drug classes was submitted to the panelists through a web-based Delphi survey. Delphi techniques are used to elicit opinion and gain consensus from a group of experts on a subject for which there is no or insufficient evidence-based information [27]. The process consists of a structured and iterative survey of at least two rounds, which continues until consensus is reached. Panelists receive feedback between each round. Delphi panels have been used extensively for generating lists of potentially inappropriate drugs in older adults [28–32].

**Fig. 1:**
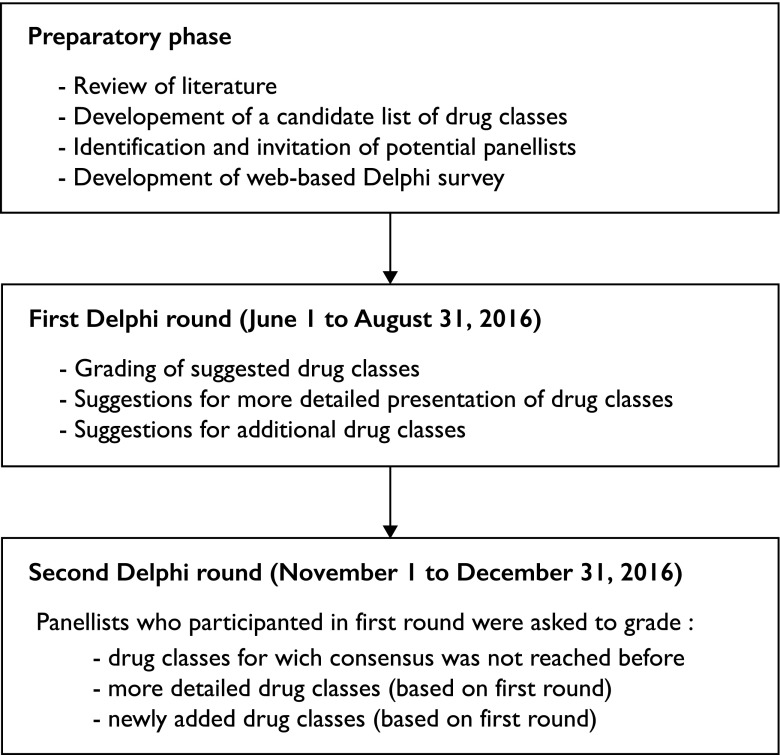
Delphi process to reach consensus about the adequateness of drug prescribing in older people at the end of life

The first Delphi round took place between June 1 and August 31, 2016 (Fig. 1). Panelists were asked to rate to what extent the prescription of different pharmacological classes included in the preliminary list seemed adequate or inadequate in older adults aged 75 years or older with an estimated life expectancy of ≤ 3 months, regardless of their underlying condition and regardless of the clinical indication of the drug. They were instructed to take into account not only the need to avoid adverse events and to ensure optimal symptom management, but also the potential futility of treatments near the end of life. We distinguished two scenarios: on the one hand, the continuation of a drug class that was previously prescribed, and on the other hand, the initiation of a new drug class. For each drug class, participants indicated their opinion on a 5-point, non-numerical Likert scale: “Always inadequate,” “Often inadequate,” “Questionable,” “Often adequate,” and “Always adequate”. They also had the possibility to suggest the inclusion of more precise drug classes in the following rounds (e.g., to break-down a large drug class into several ones), and to add drugs or drug classes that were not initially included. Participants received up to three email reminders. Consensus was considered as achieved if at least 75% of participants rated a given drug class as either “Always/often inadequate” or “Always/often adequate” for continuation and initiation, respectively. Drugs for which consensus was reached at this stage were not reviewed again during the subsequent round.

**Fig. 2:**
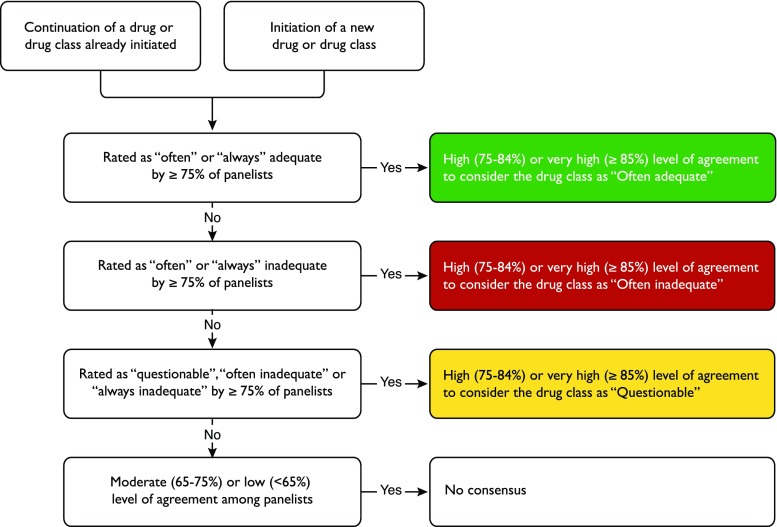
Flowchart used to determine consensus among panelists

During the second round (November 1 to December 31, 2016), panelists were provided with feedback consisting of detailed results for each item rated during round 1 as well as comments and requests from the other participants. They were asked to re-rate drug classes for which consensus was not achieved previously, and to rate drug classes or subgroups that were proposed during the first round. Results were analyzed to evaluate the level of agreement for each criterion. The categorization of drug classes is illustrated by the diagram presented in Fig. 2. Only drugs and drug classes for which the level of agreement was at least 75% were included in the final list of criteria. Drug classes with a moderate (65–74%) or low (< 65%) level of agreement are presented in Appendix Table in the ESM. The regional ethical review Board in Stockholm, Sweden, approved the study (decision no. 2015/1341-31/1).

#### Availability of data and material

The datasets generated and/or analyzed during the current study are not publicly available due to privacy issues, but are available from the corresponding author on reasonable request.

## Results

### Participating experts

A total of 58 potential participants were invited, of whom 40 (69%) agreed to be part of the consensus panel and responded in the first Delphi round. Of them, 39 also participated in the second round. As shown in Table 1, 58% of panelists were female, and 85% had at least 10 years of clinical experience. Although participants were predominantly geriatricians (33%) or palliative care physicians (30%), the panel also included seven general practitioners, seven pharmacists or clinical pharmacologists, and one palliative care psychiatrist. Participants were located in 10 different European countries.

**Table 1:** Characteristics of panelists

	*n* (%)
Total	40 (100%)
Sex	
Men	17 (43%)
Women	23 (58%)
Age	
< 45 years	16 (40%)
45–54 years	12 (30%)
55 years and older	12 (30%)
Occupation	
Geriatrician	13 (33%)
Palliative care physician	12 (30%)
General practitioner	7 (18%)
Pharmacist or pharmacologist	7 (18%)
Psychiatrist	1 (3%)
Clinical experience	
< 5 years	2 (5%)
5 to 10 years	4 (10%)
More than 10 years	34 (85%)
Country	
Belgium	1 (3%)
France	5 (13%)
Germany	3 (8%)
Italy	5 (13%)
Norway	2 (5%)
Portugal	2 (5%)
Spain	8 (20%)
Sweden	2 (5%)
Switzerland	4 (10%)
UK	8 (20%)

### Continuation of previously prescribed drug classes

During the first Delphi round, panelists reached consensus to consider the continuation of seven drug classes as “often adequate” and four drug classes as “often inadequate”. They asked for 33 suggested drug classes to be broken-down into 96 more detailed sub-classes and suggested the inclusion of 13 additional drugs in the following round (e.g., mirtazapine, haloperidol, metoclopramide). As a result, the candidate set compiled for the second Delphi round was composed of a total of 114 distinct drugs and drug classes. Consensus was achieved to consider the continuation of 9 drug classes as “often adequate,” 44 drug classes “questionable,” and 8 drug classes “often inadequate”. To facilitate the interpretation of the findings, drugs were then categorized into homogenous ATC classes, which resulted in a final list of 14 drugs or drug classes considered “often adequate,” 28 considered “questionable,” and 10 considered “often inadequate” for continuation in older adults with ≤ 3 months life expectancy (Table 2). In addition, 29 drug classes obtained only a moderate consensus (level of agreement 65 to 74%) and were therefore not included in the final set of criteria. Due to a low level of agreement (< 65%), consensus remained unachieved for 24 drug classes (Appendix Table in the ESM).

**Table 2:** Consensus criteria regarding the *continuation* of drug therapy for older adults (≥ 75 years) with an estimated life expectancy of 3 months or less

Drug or drug class (ATC code)	
Often adequate	
Butylscopolamine (A03BB01)	
Antiemetics and antinauseants (A04A)	
Drugs for constipation (A06)	
Glucocorticoids for systemic use (H02AB)	
Thyroid hormones (H03AA)	
Opioid analgesics (N02A)	
Non-opioid analgesics (N02B)	
Antiepileptics (N03)^a^	
Anxiolytics: benzodiazepines (N05BA)	
Hypnotics and sedatives: benzodiazepines (N05CD)	
Levodopa (N04BA)	
Salbutamol, inhalant (R03AC02)	
Glucocorticoids, inhalant (R03BA)	
Ipratropium, inhalant (R03BB01)	
Questionable	
Drugs for acid-related disorders, excluding PPI (A02)	
Sulfonylureas (A10BB)	
Other oral antidiabetics, excluding metformin (A10B)	
Vitamin K antagonists (B01AA)	
Unfractionated heparin (B01AB01)	
Low-dose aspirin (B01AC06)	
Other platelet aggregation inhibitors (B01AC)	
Novel oral anticoagulants (B01AE, B01AF)	
Other anticoagulants (B01AD, B01AX)	
Antianemic preparations (B03)	
Blood products (B05A)	
Digitalis glycosides (C01AA)	
Other cardiac glycosides (C01A)	
Alpha-blocker antihypertensives (C02CA, C02LE)	
Low-ceiling diuretics, thiazides (C03A)	
Low-ceiling diuretics, non-thiazides (C03B)	
Potassium-sparing agents, excluding spironolactone (C03D)	
Non-selective beta-blockers (C07AA)	
Calcium channel blockers (C08)	
Angiotensin-converting-enzyme inhibitors (C09A, C09B)	
Angiotensin II antagonists (C09C, C09D)	
Finasteride (G04CA51)	
Iodine therapy (H03C)	
Antineoplastic drugs (L01)	
Endocrine therapies (L02)	
Immunosuppressants (L04A)	
Anti-gout drugs, excluding allopurinol and colchicine (M04)	
Systemic drugs for obstructive airway diseases (R03C, R03D)	
Often inadequate	
Vitamin D (A11CC)	
Calcium supplement (A12A)	
Cardiac stimulants other than glycosides (C01C)	
Antihypertensives, excluding α-blockers (C02)	
Peripheral vasodilators (C04)	
Lipid-modifying agents (C10)	
Immunostimulants (L03A)	
Bisphosphonates (M05BA)	
Other osteoporosis drugs (M05B)	
Antidementia drugs (N06D)	

*ATC*, anatomical therapeutic chemical classification; *PPI*, proton-pump inhibitor

Level of agreement was classified as high (75–84%) or very high (≥ 85%)

^a^Excluding drugs (e.g., gabapentin, pregabalin) prescribed for the management of neuropathic pain

### Initiation of new drug classes

Consensus was achieved during the first Delphi round to consider the initiation of 4 drug classes as “often adequate” and 11 drug classes as “often inadequate” (out of the 49 included in the initial candidate set). The participants suggested that 22 drug classes should be presented at a more detailed level and asked for the inclusion of 13 additional drugs or drug classes. This resulted in a candidate set of 85 drugs and drug classes to be evaluated during the second Delphi round. Panelists reached consensus for 7 drug classes deemed “often adequate,” 39 drug classes deemed “questionable,” and 24 drug classes deemed “often inadequate”. After regrouping these items into homogenous ATC classes, the final list was composed of 10 drugs or drug classes considered “often adequate,” 23 considered “questionable,” and 23 considered “often inadequate” for initiation in older adults with ≤ 3 months life expectancy (Table 3). Among drugs for which consensus was not reached, the level of agreement between experts was moderate (65–74%) for 15 drug classes and low (< 65%) for 25 drug classes, including proton-pump inhibitors, furosemide, haloperidol, olanzapine, zopiclone, zolpidem, and selective serotonin reuptake inhibitors (see Appendix Table in the ESM).

**Table 3:** Consensus criteria regarding the *initiation* of drug therapy for older adults (≥ 75 years) with an estimated life expectancy of 3 months or less

Drug or drug class (ATC code)	
Often adequate	
Butylscopolamine (A03BB01)	
Metoclopramide (A03FA01)	
Antiemetics and antinauseants (A04A)	
Drugs for constipation (A06)	
Glucocorticoids for systemic use (H02AB)	
Opioid analgesics (N02A)	
Non-opioid analgesics (N02B)	
Clonazepam (N03AE01)	
Levetiracetam (N03AX14)	
Other antiepileptics (N03)^a^	
Anxiolytics: benzodiazepines (N05BA)	
Questionable	
Drugs for acid-related disorders, excluding PPI (A02)	
Intermediate-acting and combined insulin (A10AC, A10AD)	
Oral antidiabetics (A10B)	
Unfractionated heparin (B01AB01)	
Low molecular weight heparin (B01AB04 to B01AB11)	
Platelet aggregation inhibitors (B01AC)	
Blood products (B05A)	
Digitalis glycosides (C01AA)	
Low-ceiling diuretics (C03A, C03B)	
High-ceiling diuretics, excluding furosemide and torasemide (C03C)	
Potassium-sparing agents (C03D)	
Beta-blocking agents (C07)	
Calcium channel blockers, excluding verapamil (C08)	
Oxybutynin (G04BD04)	
Drugs for prostatic hypertrophy, excluding finasteride (G04C)	
Anti-thyroid drugs (H03B)	
Iodine therapy (H03C)	
Anti-gout medications, excluding colchicine (M04)	
Anti-Parkinson drugs, excluding levodopa (N04)	
Hypnotics and sedatives, excluding benzodiazepines (N05C)	
Tricyclic antidepressants (N06AA)^a^	
Monoamine oxidase inhibitors (N06AF-AG)	
Systemic drugs for obstructive airway diseases (R03C, R03D)	
Often inadequate	
Vitamin D (A11CC)	
Calcium supplement (A12A)	
Vitamin K antagonists (B01AA)	
Novel oral anticoagulants (B01AE, B01AF)	
Other anticoagulants (B01AD, B01AX)	
Iron preparations and erythropoietin (B03A, B03XA01)	
Vitamin B_12_ and folic acid (B03B)	
Cardiac glycosides, excluding digoxin (C01A)	
Other cardiac stimulants (C01C)	
Antihypertensives (C02)	
Peripheral vasodilators (C04)	
Verapamil (C08DA01)	
Angiotensin-converting-enzyme inhibitors (C09A, C09B)	
Angiotensin II antagonists (C09C, C09D)	
Lipid-modifying agents (C10)	
Drugs for urinary incontinence, excluding oxybutynin (G04BD)	
Finasteride (G04CA51)	
Antineoplastic drugs (L01)	
Endocrine therapies (L02)	
Immunostimulant (L03A)	
Immunosuppressants (L04A)	
Bisphosphonates and other osteoporosis drugs (M05B)	
Antidementia drugs (N06D)	

*ATC*, anatomical therapeutic chemical classification; *PPI*, proton-pump inhibitor

Level of agreement was classified as high (75–84%) or very high (≥ 85%)

^a^Excluding drugs (e.g., gabapentin, pregabalin, amitriptyline) prescribed for the management of neuropathic pain

## Discussion

In this study, 40 European experts in geriatrics, palliative medicine, general practice, pharmacy, and clinical pharmacology from 10 different countries provided their professional opinion drug prescribing in older adults aged 75 years or older with an estimated life expectancy of ≤ 3 months. Panelists were given the opportunity to differentiate between the continuation and the initiation of drug therapy. Based on these results, we developed a consensus list of adequate, questionable, and inadequate drugs for older adults near the end of life.

Drug classes considered as *often adequate* by a qualified majority of panelists (≥ 75% agreement) are mostly prescribed for symptom management and comfort, e.g., analgesics, antinauseants, anxiolytics, drugs for constipation, levodopa. This is in line with the palliative goals of care often established near the end of life, and medications classified as often adequate are in fact highly congruent with the list of essential medicines in palliative care established by the World Health Organization in collaboration with the International Association for Hospice and Palliative Care (IAHPC) [33]. Consensus criteria for drugs of *questionable* benefit are for a large part centered around treatments used for the control of non-life-threatening comorbidities or for the secondary prevention of cardiovascular diseases, e.g., platelet aggregation inhibitors and oral antidiabetics. This category also contains drugs commonly prescribed to counteract the side effects of other treatment—a phenomenon known as the prescription cascade. Finally, drug classes categorized as *often inadequate* include a majority of drug treatments used for the long-term prevention of chronic diseases (e.g., vitamin D, statins).

Our approach does not assume that certain drugs are inherently futile or unnecessary at the end of life. Participating experts were asked to provide their probabilistic opinion based on their clinical experience and scientific knowledge rather than an all-embracing judgment with no room for uncertainty or case-by-case variability. For example, panelists reached consensus to consider that continuing antidementia drugs is *often inadequate* near the end of life, even though it could be justified in specific situations. Conversely, even if one can think of individual patients for whom opioid analgesics would not provide any substantial benefit at the end of life, in a majority of cases it is adequate. Consensus-based criteria are useful to provide guidance and to encourage benchmarking, but decisions regarding the continuation or discontinuation of drug treatments should be tailored at the bedside to address the specific needs and preferences of each individual patient. Recognizing the need for a personalized assessment of drug therapy is essential to avoid the pitfalls of one-size-fits-all clinical standards. Thus, panelists were given the opportunity to express a consensus about the *questionable* nature of the decision to continue or initiate drug treatments for older adults with very limited life expectancy. We have identified a number of drug classes that may very well be adequate at the end of life, but that should be systematically questioned beforehand and not result from routine-based prescribing practices. This approach is well in line with the *Choosing Wisely* campaign, in which medical societies have identified tests, procedures, and treatments that are often used inappropriately and that both physicians and patients should question [34, 35].

In the present study, drugs were considered for use among older adults with a maximum of 3 months of life expectancy. In contrast, the STOPPFrail criteria [22] were developed to identify inappropriate drugs among older adults with poor 1-year prognosis, thus broadening the perspective to a substantially longer period of time before death. Physicians and patients typically overestimate the remaining life expectancy, and the accuracy of survival predictions tends to decrease as the timeframe increases [26, 36, 37]. Therefore, determining whether the time-to-benefit of medications is shorter than life expectancy is more difficult for patients who might live for another 12–18 months than for persons whose life expectancy is estimated to be 1–3 months. Our focus on the very end of life may reduce the prognostic uncertainty, and thus enhance the applicability of the proposed lists of drugs in routine clinical practice.

The *continuation* and the *initiation* of drug therapy near the end of life pose different challenges in the daily clinical practice. From a pharmacological perspective, the discontinuation of specific drug classes (e.g., antidepressants) is known to trigger withdrawal symptoms and adverse effects that may cause discomfort and affect quality of life at the end of life. Cessation of these drugs requires time-consuming tapering of dosage and a careful monitoring of symptoms [14, 38, 39]. This process implies the availability of a physician at the bedside, which is not always feasible in the everyday care setting. Moreover, stopping drug treatments is often perceived by prescribers as an active decision that can lead to serious adverse consequences, which creates a strong incentive against deprescribing out of fear that it may cause harm. In comparison, the decision not to initiate a new drug treatment is widely considered as an omission rather than as an act. As a result, physicians have been found to perceive the limitation of treatments as less likely to result in negative outcomes than withdrawal [40]. Acknowledging that the degree of appropriateness of a prescription depends on whether therapy is being *started* or *continued* can increase the acceptability of our list of inadequate drugs by clinicians. It can also serve as a reminder that good prescribing is not only about knowing when to *start* and when to *stop* treating patients, but also about knowing when *not to start* in the first place.

Consensus remained unachieved for some very commonly prescribed drug classes. Neither the continuation nor the initiation of proton-pump inhibitors (PPI) gathered a consensual opinion among panelists. This lack of agreement mirrors the existence of conflicting positions regarding the use of these drugs near the end of life. While Holmes et al. considered PPIs to be “sometimes appropriate” for older adults with advanced dementia and limited life expectancy [18], De Schreye et al. came to the opposite conclusion and considered all gastric protectors to be inappropriate during the last 6 months of life [41]. Participating experts also did not reach consensus regarding the continuation and the initiation of selective serotonin-reuptake inhibitors (SSRI). This was unexpected, since citalopram and sertraline are recommended as first-line treatment options for depression in palliative care patients and have fewer side effects than other antidepressants, lower risks of drug-drug interactions, and a shorter time-to-benefit [42, 43]. Similarly, consensus remained unachieved for haloperidol, an antipsychotic often prescribed in palliative care practice for the management of delirium [44, 45]. Although 67% of panelists considered the continuation of haloperidol to be “often adequate,” no majority opinion emerged regarding initiation. This may be explained by the lack of evidence regarding the effectiveness of haloperidol for the management of delirium compared with other alternatives (e.g., quetiapine, olanzapine, risperidone) or even with placebo. Recent randomized controlled trials showed that haloperidol did not reduce delirium and resulted in significantly higher extrapyramidal symptoms [46, 47].

The present study present several limitations. First, although consensus methods are widely used to inform clinical practice in the absence of robust empirical data, expert judgment ranks low in the hierarchy of evidence. However, our study was designed to mitigate potential biases, as recommended by Jünger et al. [48]. The panel was larger than for previously published criteria, and composition was balanced with regard to age, occupations, and countries (thus reducing the homogeneity of clinical practices and prescribing attitudes). Consensus attainment was defined a priori based on a 75% agreement cutoff, providing a rather conservative set of criteria for each category. We also made sure that the material sent to participating experts would not directly or indirectly influence their opinion. The list of participants was kept undisclosed during the Delphi survey to prevent cross-contamination among panelists. Second, although it can be argued that implicit criteria (e.g., *Medication Appropriateness Index* [49]) are clinically more relevant, they cannot be operationalized in large drug prescription databases. Explicit criteria inevitably reduce the complexity of personal, clinical, and ethical considerations that surround the decision to prescribe a specific treatment. However, it is unrealistic to expect data with individualized assessments in large (sometimes country-wide) study cohorts of older adults at the end of life. Third, we asked panelists to grade the appropriateness of drug treatments regardless of their clinical indications although many drug classes may be used for specific health problems near the end of life. This probably explains why some drug classes did not reach a sufficiently high level of agreement. Fourth, our criteria are based on the expert judgment of European clinicians, and may therefore reflect cultural preferences or prescribing attitudes that are specific to European countries. Further studies should therefore investigate whether they also generate consensus in other geographical areas. Also, some adjustments may be necessary to apply these criteria to non-European care settings since the availability of specific drugs varies across national markets. Finally, it should be remembered that our criteria have not been developed to replace the clinical judgment of prescribers. They should instead to be used to encourage patient–physician discussions about the benefits and risks of drug treatments near the end of life.

## Conclusion

Considering the lack of robust evidence from randomized clinical trials and well-designed observational studies, our criteria can provide guidance to rationalize drug prescribing for older adults near the end of life. These criteria also offer a standardized framework for future studies investigating the prevalence and the adverse outcomes associated with the use of questionable or inadequate drugs at the end of life.

## Electronic supplementary material

ESM 1 (DOCX 256 kb)
