# Supplementary material for: Adequate, questionable, and inadequate drug prescribing for older adults at the end of life: a European expert consensus
Source: Eur J Clin Pharmacol. 2018 Jun 23;74(10):1333–42. doi: 10.1007/s00228-018-2507-4 (PMC6132505; doi:10.1007/s00228-018-2507-4)
Supplement: Supplementary file 1 — (DOCX 256 kb) [file 228_2018_2507_MOESM1_ESM.docx]

**Appendix Table - Continuation and initiation of drug therapy in older adults (≥75 years) with an estimated life expectancy of 3 months or less: level of agreement and consensus among European experts**

| **Drug or drug class (ATC code)** | **Continuation of drug therapy** | | |  | **Initiation of drug therapy** | | |
| --- | --- | --- | --- | --- | --- | --- | --- |
|  | **Panelists’ opinion** | | **Level of agreement*** |  | **Panelists’ opinion** | | **Level of agreement*** |
|  | Majority opinion | No. / No/ total (%) |  |  | Majority opinion | No. / No/ total (%) |  |
| **A - ALIMENTARY TRACT AND METABOLISM** |  |  |  |  |  |  |  |
| Proton-Pump Inhibitors (A02BC) | – | – | Low |  | – | – | Low |
| Other drugs for acid-related disorders (A02) |  Questionable | 35 / 39 (90%) | Very high |  |  Questionable | 34 / 39 (87%) | Very high |
| Butylscopolamine (A03BB01) |  Often adequate | 32 / 39 (82%) | High |  |  Often adequate | 31 / 39 (79%) | High |
| Metoclopramide (A03FA01) |  Often adequate | 28 / 39 (72%) | Moderate |  |  Often adequate | 31 / 39 (79%) | High |
| Antiemetics and antinauseants (A04A) |  Often adequate | 36 / 40 (90%) | Very high |  |  Often adequate | 36 / 40 (90%) | Very high |
| Drugs for constipation (A06) |  Often adequate | 35 / 40 (88%) | Very high |  |  Often adequate | 37 / 40 (93%) | Very high |
| Fast-acting insulin (A10AB) |  Often adequate | 24 / 37 (65%) | Moderate |  | – | – | Low |
| Intermediate-acting insulin (A10AC) | – | – | Low |  |  Questionable | 28 / 37 (76%) | High |
| Combined insulin (A10AD) | – | – | Low |  |  Questionable | 31 / 37 (84%) | High |
| Long-acting insulin (A10AE) | – | – | Low |  |  Questionable | 25 / 37 (68%) | Moderate |
| Metformin and analogues (A10BA) |  Questionable | 26 / 37 (70%) | Moderate |  |  Questionable | 32 / 37 (86%) | Very high |
| Sulfonylurea (A10BB) |  Questionable | 31 / 37 (84%) | High |  |  Questionable | 33 / 37 (89%) | Very high |
| Gliptins (A10BH) |  Questionable | 31 / 36 (86%) | Very high |  |  Questionable | 34 / 36 (94%) | Very high |
| Other oral antidiabetics (A10B) |  Questionable | 34 / 36 (94%) | Very high |  |  Questionable | 35 / 36 (97%) | Very high |
| Vitamin D (A11CC) |  Often inadequate | 33 / 40 (83%) | High |  |  Often inadequate | 34 / 40 (85%) | Very high |
| Calcium supplement (A12A) |  Often inadequate | 34 / 40 (85%) | Very high |  |  Often inadequate | 35 / 40 (88%) | Very high |
| **B - BLOOD AND BLOOD FORMING ORGANS** |  |  |  |  |  |  |  |
| Vitamin K antagonists (B01AA) |  Questionable | 30 / 38 (79%) | High |  |  Often inadequate | 31 / 38 (82%) | High |
| Unfractionated heparin (B01AB01) |  Questionable | 34 / 37 (92%) | Very high |  |  Questionable | 33 / 37 (89%) | Very high |
| Low molecular weight heparin (B01AB01 to 11) |  Questionable | 27 / 37 (73%) | Moderate |  |  Questionable | 28 / 37 (76%) | High |
| Clopidogrel (B01AC04) |  Questionable | 35 / 38 (92%) | Very high |  |  Questionable | 35 / 38 (92%) | Very high |
| Low-dose aspirin (B01AC06) |  Questionable | 32 / 38 (84%) | High |  |  Questionable | 35 / 38 (92%) | Very high |
| Other Platelet aggregation inhibitors (B01AC) |  Questionable | 36 / 38 (95%) | Very high |  |  Questionable | 36 / 38 (95%) | Very high |
| Novel oral anticoagulants (B01AE, B01AF) |  Questionable | 31 / 38 (82%) | High |  |  Often inadequate | 32 / 37 (86%) | Very high |
| Other anticoagulants (B01AD, B01AX) |  Questionable | 35 / 37 (95%) | Very high |  |  Often inadequate | 33 / 37 (89%) | Very high |
| Iron preparations (B03A) |  Questionable | 38 / 39 (97%) | Very high |  |  Often inadequate | 32 / 39 (82%) | High |
| Vitamin B12 and Folic acid (B03B) |  Questionable | 39 / 39 (100%) | Very high |  |  Often inadequate | 34 / 39 (87%) | Very high |
| Erythropoietin (B03XA01) |  Questionable | 38 / 39 (97%) | Very high |  |  Often inadequate | 32 / 39 (82%) | High |
| Erythrocytes (B05AX01) |  Questionable | 34 / 39 (87%) | Very high |  |  Questionable | 35 / 39 (90%) | Very high |
| Thrombocytes (B05AX02) |  Questionable | 35 / 39 (90%) | Very high |  |  Questionable | 35 / 39 (90%) | Very high |
| Other blood products (B05A) |  Questionable | 38 / 39 (97%) | Very high |  |  Questionable | 39 / 39 (100%) | Very high |
| **C - CARDIOVASCULAR SYSTEM** |  |  |  |  |  |  |  |
| Digitalis glycosides (C01AA) |  Questionable | 30 / 39 (77%) | High |  |  Questionable | 34 / 39 (87%) | Very high |
| Other cardiac glycosides (C01A) |  Questionable | 35 / 39 (90%) | Very high |  |  Often inadequate | 30 / 39 (77%) | High |
| Adrenergic and dopaminergic agents (C01CA) |  Often inadequate | 34 / 39 (87%) | Very high |  |  Often inadequate | 37 / 39 (95%) | Very high |
| Other cardiac stimulants (C01C) |  Often inadequate | 36 / 39 (92%) | Very high |  |  Often inadequate | 37 / 39 (95%) | Very high |
| α-blocker antihypertensives (C02CA, C02LE) |  Questionable | 36 / 39 (92%) | Very high |  |  Often inadequate | 33 / 40 (83%) | High |
| Other antihypertensives (C02) |  Often inadequate | 33 / 39 (85%) | Very high |  |  Often inadequate | 32 / 40 (80%) | High |
| Diuretics, thiazides (C03A) |  Questionable | 31 / 39 (79%) | High |  |  Questionable | 35 / 39 (90%) | Very high |
| Diuretics, non-thiazides (C03B) |  Questionable | 35 / 39 (90%) | Very high |  |  Questionable | 36 / 39 (92%) | Very high |
| Furosemide (C03CA01) |  Often adequate | 26 / 39 (67%) | Moderate |  | – | – | Low |
| Torasemide (C03CA04) | – | – | Low |  |  Questionable | 26 / 39 (67%) | Moderate |
| Other high-ceiling diuretics (C03C) |  Questionable | 27 / 39 (69%) | Moderate |  |  Questionable | 31 / 39 (79%) | High |
| Spironolactone (C03DA01) |  Questionable | 26 / 39 (67%) | Moderate |  |  Questionable | 33 / 39 (85%) | Very high |
| Other potassium-sparing agents (C03D) |  Questionable | 32 / 39 (82%) | High |  |  Questionable | 38 / 39 (97%) | Very high |
| Peripheral vasodilators (C04) |  Often inadequate | 34 / 40 (85%) | Very high |  |  Often inadequate | 36 / 40 (90%) | Very high |
| Non-selective beta-blockers (C07AA) |  Questionable | 34 / 39 (87%) | Very high |  |  Questionable | 38 / 39 (97%) | Very high |
| Selective beta-blockers (C07AB) |  Questionable | 28 / 39 (72%) | Moderate |  |  Questionable | 33 / 39 (85%) | Very high |
| Alpha and beta blocking agents (C07AG) |  Questionable | 29 / 39 (74%) | Moderate |  |  Questionable | 35 / 39 (90%) | Very high |
| Amlodipine (C08CA01) |  Questionable | 33 / 39 (85%) | Very high |  |  Questionable | 39 / 39 (100%) | Very high |
| Verapamil (C08DA01) |  Questionable | 34 / 39 (87%) | Very high |  |  Often inadequate | 31 / 39 (79%) | High |
| Other calcium channel blockers (C08) |  Questionable | 35 / 39 (90%) | Very high |  |  Questionable | 39 / 39 (100%) | Very high |
| Ramipril (C09AA03) |  Questionable | 31 / 39 (79%) | High |  |  Often inadequate | 33 / 39 (85%) | Very high |
| Lisinopril (C09AA05) |  Questionable | 32 / 39 (82%) | High |  |  Often inadequate | 34 / 39 (87%) | Very high |
| Other ACE inhibitors (C09AA, C09B) |  Questionable | 32 / 39 (82%) | High |  |  Often inadequate | 34 / 39 (87%) | Very high |
| Angiotensin II antagonists (C09C, C09D) |  Questionable | 33 / 39 (85%) | Very high |  |  Often inadequate | 34 / 39 (87%) | Very high |
| Lipid-modifying agents (C10) | Often inadequate | 37 / 40 (93%) | Very high |  | Often inadequate | 38 / 40 (95%) | Very high |
| **G – GENITO-URINARY SYSTEM AND SEX HORMONES** | |  |  |  |  |  |  |
| Oxybutynin (G04BD04) | Questionable | 28 / 39 (72%) | Moderate |  | Questionable | 34 / 39 (87%) | Very high |
| Other drugs for urinary incontinence (G04BD) | Questionable | 29 / 39 (74%) | Moderate |  | Often inadequate | 30 / 39 (77%) | Very high |
| Finasteride (G04CA51) | Questionable | 30 / 39 (77%) | High |  | Often inadequate | 32 / 39 (82%) | High |
| Other drugs for prostatic hypertrophy (G04C) | Questionable | 29 / 39 (74%) | Moderate |  | Questionable | 38 / 39 (97%) | Very high |
| **H - SYSTEMIC HORMONAL PREPARATIONS** |  |  |  |  |  |  |  |
| Mineralocorticoids (H02AA) | – | – | Low |  | – | – | Low |
| Glucocorticoids (H02AB) | Often adequate | 32 / 39 (82%) | High |  | Often adequate | 31 / 39 (79%) | High |
| Thyroid hormones (H03A) | Often adequate | 30 / 39 (77%) | High |  | Questionable | 27 / 39 (69%) | Moderate |
| Anti-thyroid drugs (H03B) | – | – | Low |  | Questionable | 30 / 39 (77%) | High |
| Iodine therapy (H03C) | Questionable | 32 / 39 (82%) | High |  | Questionable | 37 / 39 (95%) | Very high |
| **L - ANTINEOPLASTIC AND IMMUNOMODULATING AGENTS** | |  |  |  |  |  |  |
| Antineoplastic drugs (L01) | Questionable | 37 / 39 (95%) | Very high |  | Often inadequate | 34 / 39 (87%) | Very high |
| Progestogens (L02AB) | Questionable | 35 / 39 (90%) | Very high |  | Often inadequate | 34 / 39 (87%) | Very high |
| GnRH analogues / LHRH agonists (L02AE) | Questionable | 35 / 39 (90%) | Very high |  | Often inadequate | 35 / 39 (90%) | Very high |
| Anti-estrogens (L02BA) | Questionable | 33 / 39 (85%) | Very high |  | Often inadequate | 35 / 39 (90%) | Very high |
| Aromatase inhibitors (L02BG) | Questionable | 33 / 39 (85%) | Very high |  | Often inadequate | 35 / 39 (90%) | Very high |
| Other endocrine therapies (L02) | Questionable | 36 / 39 (92%) | Very high |  | Often inadequate | 35 / 39 (90%) | Very high |
| Immunostimulants (L03A) | Often inadequate | 36 / 39 (92%) | Very high |  | Often inadequate | 33/ 40 (83%) | Very high |
| TNF-α inhibitors (L04AB) | Questionable | 36 / 39 (92%) | Very high |  | Often inadequate | 36 / 39 (92%) | Very high |
| Other immunosuppressants (L04A) | Questionable | 37 / 39 (95%) | Very high |  | Often inadequate | 34 / 39 (87%) | Very high |
| **M - MUSCULO-SKELETAL SYSTEM** |  |  |  |  |  |  |  |
| Acetic acid derivatives (M01AB) | – | – | Low |  | – | – | Low |
| Propionic acid derivatives (M01AE) | – | – | Low |  | Questionable | 27 / 39 (69%) | Moderate |
| Coxibs (M01AH) | Questionable | 27 / 39 (69%) | Moderate |  | Questionable | 29 / 39 (74%) | Moderate |
| Baclofen (M03BX01) | Often adequate | 27 / 39 (69%) | Moderate |  | – | – | Low |
| Other muscle relaxants (M03) | – | – | Low |  | – | – | Low |
| Allopurinol (M04AA01) | Questionable | 29 / 39 (74%) | Moderate |  | Questionable | 36 / 39 (92%) | Very high |
| Colchicine (M04AC01) | Questionable | 29 / 39 (74%) | Moderate |  | Questionable | 29 / 39 (74%) | Moderate |
| Other anti-gout medications (M04) | Questionable | 32 / 39 (82%) | High |  | Questionable | 36 / 39 (92%) | Very high |
| Bisphosphonates (M05BA) | Often inadequate | 30 / 39 (77%) | High |  | Often inadequate | 34 / 40 (85%) | Very high |
| Other anti-osteoporosis drugs (M05B) | Often inadequate | 35 / 39 (90%) | Very high |  | Often inadequate | 34 / 40 (85%) | Very high |
| **N - NERVOUS SYSTEM** |  |  |  |  |  |  |  |
| Lidocaine for neuropathic pain (N01BB02, N01BB52) | – | – | Low |  | – | – | Low |
| Ketamine (N01AX03) | – | – | Low |  | – | – | Low |
| Nitrous oxide (N01AX13) | Questionable | 27 / 39 (69%) | Moderate |  | Questionable | 29 / 39 (74%) | Moderate |
| Opioids (N02A) | Often adequate | 37 / 40 (93%) | Very high |  | Often adequate | 38 / 40 (95%) | Very high |
| Non-opioid analgesics (N02B) | Often adequate | 36 / 40 (90%) | Very high |  | Often adequate | 36 / 40 (90%) | Very high |
| Clonazepam (N03AE01) | Often adequate | 32 / 40 (80%) | High |  | Often adequate | 33 / 39 (85%) | Very high |
| Gabapentin for neuropathic pain (N03AX12) | Often adequate | 29 / 39 (74%) | Moderate |  | Often adequate | 26 / 39 (67%) | Moderate |
| Levetiracetam (N03AX14) | Often adequate | 32 / 40 (80%) | High |  | Often adequate | 34 / 39 (87%) | Very high |
| Pregabalin for neuropathic pain (N03AX16) | Often adequate | 28 / 39 (72%) | Moderate |  | – | – | Low |
| Other antiepileptics (N03) | Often adequate | 32 / 40 (80%) | High |  | Often adequate | 31 / 39 (79%) | High |
| Levodopa (N04BA) | Often adequate | 33 / 39 (85%) | Very high |  | Questionable | 28 / 39 (72%) | Moderate |
| Dopamine agonists (N04BC) | Often adequate | 28 / 39 (72%) | Moderate |  | Questionable | 33 / 39 (85%) | Very high |
| Other anti-Parkinson drugs (N04) | Often adequate | 27 / 39 (69%) | Moderate |  | Questionable | 33 / 39 (85%) | Very high |
| Haloperidol (N05AD01) | Often adequate | 26 / 39 (67%) | Moderate |  | – | – | Low |
| Levomepromazine (N05AA02) | – | – | Low |  | – | – | Low |
| Other antipsychotics (N05A) | – | – | Low |  | – | – | Low |
| Anxiolytics: benzodiazepines (N05BA) | Often adequate | 33 / 39 (85%) | Very high |  | Often adequate | 34 / 39 (87%) | Very high |
| Other anxiolytics (N05B) | – | – | Low |  | – | – | Low |
| Hypnotics and sedatives: benzodiazepines (N05CD) | Often adequate | 34 / 39 (87%) | Very high |  | Often adequate | 29 / 39 (74%) | Moderate |
| Other hypnotics and sedatives (N05C) | Questionable | 26 / 39 (67%) | Moderate |  | Questionable | 31 / 39 (79%) | High |
| Olanzapine (N05AH03) | – | – | Low |  | – | – | Low |
| Zopiclone (N05CF01) | Often adequate | 28 / 39 (72%) | Moderate |  | – | – | Low |
| Zolpidem (N05CF02) | Often adequate | 28 / 39 (72%) | Moderate |  | – | – | Low |
| Eszopiclone (N05CF04) | – | – | Low |  | – | – | Low |
| Melatonin (N05CH01) | Questionable | 26 / 39 (67%) | Moderate |  | Questionable | 29 / 39 (74%) | Moderate |
| Tricyclic antidepressants for neuropathic pain (N06AA) | – | – | Low |  | – | – | Low |
| Tricyclic antidepressants for depression (N06AA) | – | – | Low |  | Questionable | 32 / 39 (82%) | High |
| Monoamine oxidase inhibitors (N06AF-AG) | Often adequate | 28 / 39 (72%) | Moderate |  | Questionable | 38 / 39 (97%) | Very high |
| Selective serotonin reuptake inhibitors (N06AB) | – | – | Low |  | – | – | Low |
| Trazodone (N06AX05) | – | – | Low |  | – | – | Low |
| Mirtazapine (N06AX11) | – | – | Low |  | – | – | Low |
| Venlafaxine for neuropathic pain (N06AX16) | – | – | Low |  | – | – | Low |
| Duloxetine for neuropathic pain (N06AX21) | – | – | Low |  | – | – | Low |
| Anticholinesterases (N06DA) | Often inadequate | 31 / 39 (79%) | High |  | Often inadequate | 36 / 40 (90%) | Very high |
| Memantine (N06DX) | Often inadequate | 31 / 39 (79%) | High |  | Often inadequate | 36 / 40 (90%) | Very high |
| Methadone (N07BC02) | – | – | Low |  | – | – | Low |
| **R - RESPIRATORY SYSTEM** |  |  |  |  |  |  |  |
| Salbutamol, inhalant (R03AC02) | Often adequate | 31 / 39 (79%) | High |  | Often adequate | 26 / 39 (67%) | Moderate |
| Glucocorticoids, inhalant (R03BA) | Often adequate | 31 / 39 (79%) | High |  | Often adequate | 27 / 39 (69%) | Moderate |
| Ipratropium, inhalant (R03BB01) | Often adequate | 33 / 39 (85%) | Very high |  | Often adequate | 28 / 39 (72%) | Moderate |
| Other inhalants for COPD (R03B) | Often adequate | 27 / 39 (69%) | Moderate |  | – | – | Low |
| Leukotriene receptor antagonists (R03DC) | Questionable | 31 / 39 (79%) | High |  | Questionable | 38 / 39 (97%) | Very high |
| Other systemic drugs for COPD (R03C, R03D) | Questionable | 31 / 39 (79%) | High |  | Questionable | 38 / 39 (97%) | Very high |
| Cyclizine (R06AE03) | Questionable | 27 / 39 (69%) | Moderate |  | Questionable | 26 / 39 (67%) | Moderate |

** The level of agreement among panelists was classified as low (<65%), moderate (65-74%), high (75-84%), and very high (≥85%). Only criteria with high or very high levels of agreement were included in the final set.*

**Abbreviations:**

ATC, Anatomical Therapeutic Chemical classification

ACE, angiotensin-converting-enzyme inhibitor

COPD, Chronic Obstructive Pulmonary Disease

**Notes:** Panelists were asked to grade the adequateness of continuing/initiating drugs regardless of their clinical indication. However, during the second round panelists requested that drugs used for the management of neuropathic pain be considered separately: gapabentin (ATC N03AX12), pregabalin (N03AX16), lidocaine (N01BB02), duloxetine (N06AX21), venlafaxine (N06AX16) and tricyclic antidepressants (specifically amitriptyline, imipramine, and clomipramine).
